# Expressions of Fib, IL-12 in Serum of Neonatal Necrotizing Enterocolitis and Their Correlation with Clinicopathological Features

**DOI:** 10.18502/ijph.v49i8.3874

**Published:** 2020-08

**Authors:** Yiyu YIN, Xiaole WU, Huaxin ZOU, Shixian LI, Zhenfang QIN, Tengfei ZHANG, Junhua CAO

**Affiliations:** 1. Department of General Surgery, Xuzhou Children’s Hospital, Xuzhou Medical University, Xuzhou 221006, P.R. China; 2. Department of Anesthesiology, Xuzhou Children’s Hospital, Xuzhou Medical University, Xuzhou 221006, P.R. China; 3. Department of Emergency Medicine, Xuzhou Children’s Hospital, Xuzhou Medical University, Xuzhou 221006, P.R. China

**Keywords:** Interleukin-12, Neonatal necrotizing enterocolitis, Clinicopathological features, Erythrocyte, Platelet

## Abstract

**Background:**

To investigate the expressions of fibrinogen (Fib) and Interleukin-12 (IL-12) in serum of neonatal necrotizing enterocolitis (NEC), and to analyze the correlation between the two and their relationship with clinicopathological features.

**Methods:**

Forty two children with NEC treated in Xuzhou Children’s Hospital, Xuzhou Medical University Xuzhou, China from 2016–2019 were selected as an observation group and 40 children who underwent physical examination at the same period as a control group. The expression levels of Fib and IL-12 in the serum of two groups were detected by ELISA. The correlation between Fib and IL-12 in the observation group and the correlation among the expressions of Fib, IL-12, the clinicopathological features and common examination indexes of the children with NEC were investigated by Pearson correlation analysis.

**Results:**

The levels of Fib and IL-12 in the serum of the children in observation group were significantly higher than those in the control group were (*P*<0.05). There was a significant positive correlation between the levels of Fib and IL-12 in the serum of the children in observation group (*P*<0.05). The expression levels of Fib, IL-12 were not significantly correlated with sex and age of NEC children, but correlated with vomiting, diarrhea, bloody stool and bradycardia in NEC children (*P*<0.05). Fib and IL-12 were positively correlated with erythrocyte level (*P*<0.05) and negatively correlated with platelet level.

**Conclusion:**

The expressions of Fib and IL-12 in the serum of NEC children can objectively predict the severity of NEC.

## Introduction

Neonatal necrotizing enterocolitis (NEC) is an acquired gastrointestinal disease with an incidence of 7%–21% and a fatality rate of about 20%, especially for premature infants. With the development of neonatal intensive care and treatment technology of perinatal doctors, the survival rate of premature infants and low birth weight infants increased, and the mortality of NEC children increased significantly (1–4). There is usually a positive correlation between gestational age and birth weight. Children with low birth weight and premature infants are the subjects with high incidence of NEC. If they are not treated in time and effectively, the death rate will be increased (5–8). Short bowel syndromes, such as the decrease of the absorption area of small intestine, the absorption disorder of nutrients, and the difficulty of treatment of diarrhea, are common after NEC operation for different reasons. NEC accounts for 35% of the causes of short bowel syndrome in neonatal intensive care (9, 10), which is a serious threat to the life and health of newborns. Therefore, it is very important to predict the condition of NEC in the early stage, and it becomes urgency.

Active intestinal inflammation can activate coagulation and fibrolysis cascade reaction, resulting in hypercoagulation and high fibrinolysis (11). Fib is an acute inflammatory reactant associated with thrombosis, and the level of Fib increases during inflammation (12). The IL-12 cytokine is an isomer functional cell regulator (13) with infectious and autoimmune disease immunity. IL-12 cytokines had anti-inflammatory and proinflammatory function model, which affected the inflammatory reaction process (14).

In this study, the expression levels of Fib and IL-12 were used to predict the severity of NEC in order to provide the direction for the diagnosis and treatment of NEC and to improve the prognosis.

## Materials and Methods

Altogether 42 children with NEC admitted to Xuzhou Children’s Hospital, Xuzhou Medical University Xuzhou, China from 2016 to 2019 were selected as an observation group, and 40 healthy children undergoing physical examination as a control group. There were 48 male children and 34 female children with the average age of (17.4±5.7) days, the average gestational age of (35.4±2.6) weeks, and the average body mass of (1846±616) grams. There was no significant difference in age, gestational age and body mass between the two groups but a significant difference in sex, vomiting and diarrhea (*P* < 0.05) (Table 1).

**Table 1:** Comparison of general information

***Group***	***Observationgroup(n=42)***	***Control group (n=40)***	***X^2^/t***	***P***
Sex			3.919	0.048
Male	29(69.05)	19(47.50)		
Female	13(30.95)	21(52.50)		
Age (days)	17.1±5.8	17.5±5.6	0.318	0.752
Gestational age (weeks)	35.2±2.8	35.5±3.0	0.468	0.641
Body mass(g)	1838±618	1848±621	0.073	0.942
Vomiting			11.711	<0.001
Yes	13(30.95)	1(2.50)		
No	29(69.05)	39(97.50)		
Diarrhea			9.988	0.002
Yes	17(40.48)	4(10.00)		
No	25(59.52)	36(90.00)		
Blood stool			16.081	<0.001
Yes	14(33.33)	0		
No	28(66.67)	40(100.00)		

### Inclusion criteria

Neonates diagnosed as necrotizing enterocolitis by pathological examination.

### Exclusion criteria

1) Newborns weighing less than 1 kg; 2) Children with congenital heart disease, congenital intestinal malformation and other congenital diseases; 3) Children with severe neonatal asphyxia; 4) Children complicated with severe intrauterine infection; 5) Children with related genetic diseases of the digestive system.

All the families of the children agreed to participate in the experiment and signed informed consent forms. This experiment was approved by the Medical Ethics Committee of Xuzhou Children’s Hospital, Xuzhou Medical University.

### Experimental Reagents and Materials

ELISA Kit (Bangyi Biotechnology Co., Ltd., Shanghai, China); anti-Fib antibody (Guangrui Biological Technology Co., Ltd., Shanghai, China); anti-IL-12 antibody (Abcam Trading Co., Ltd., Shanghai, China); plasma separator (Beckman Inc., America); enzymatic marker (Thermo Fisher Scientific Inc, China).

### Test Method

All the children were fasting for 4 hours and deprived of water for 2 hours before the experiment. A 5-mL sample of fasting venous blood was collected in the morning for blood routine, Fib and IL-12 detection. The blood was centrifuged by serum separation centrifuge at 3000 r/min for 6 min to collect the serum. ELISA was used for determination of serum Fib. Two hundred ul of serum samples from the study group and the control group were added to the pore plate coated with anti-Fib antibody. At the same time, the standard and blank holes were set. After 70 min reaction at room temperature, the reaction solution was dried and rinsed for 3 times. Then 100 μl of chromogenic agent was added for color reaction at 37 °C for 20 min. The average optical density of each hole was recorded by putting 100 μl of mixture into the enzyme standard instrument at 500 nm wavelength. The IL-12 level detection was as the same as the above method. The experiment was carried out strictly according to the instructions of ELISA kit.

### Observation Indexes

1. Fib and IL-12 levels in Children of the observation group and the control group were comparatively analyzed.
2. Correlation analysis was performed on the levels of Fib and IL12 in children in the observation group.
3. Relationship between clinicopathological features and expressions of Fib, IL-12 in children with NEC was observed.
4. Correlation between the expressions of Fib, IL-12 and the common clinical examination indexes in children with NEC was analyzed.

### Statistical Methods

The SPSS20.0 (NDTimes Technology Co., Ltd., Beijing, China) software was used to analyze statistically the collected data. Data were analyzed using the chi-squared test, and the measured data were expressed as the mean ± standard deviation. T test was used to analyze the two groups. Pearson test was used for correlation analysis. GraphPad Prism 8 was used to plot the figures in this experiment. A value of *P*<0.05 was considered as indicating a statistically significant difference between two groups.

## Results

**Fig. 1:**
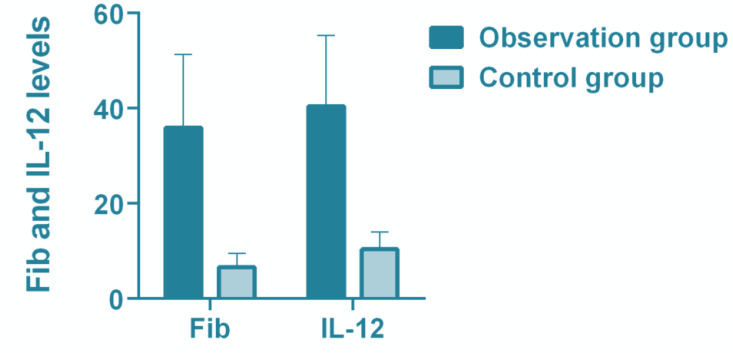
Comparative analysis of Fib, IL-12 levels between the observation group and the control group

The serum Fib in the observation group was significantly higher than that in the control group (*P*<0.05). The serum IL-12 in the observation group was significantly higher than that in the control group (*P*<0.05) (Table 2, Fig. 1).

**Table 2:** Comparison of Fib, IL-12 levels between the observation group and the control group

***Variable***	***Observation group (n=42)***	***Control group (n=40)***	***t***	***P***
Fib(ng/L)	35.93±15.37	6.63±2.83	11.860	<0.001
IL-12(ng/L)	40.43±14.82	10.37±3.65	12.470	<0.001

**Fig. 2:**
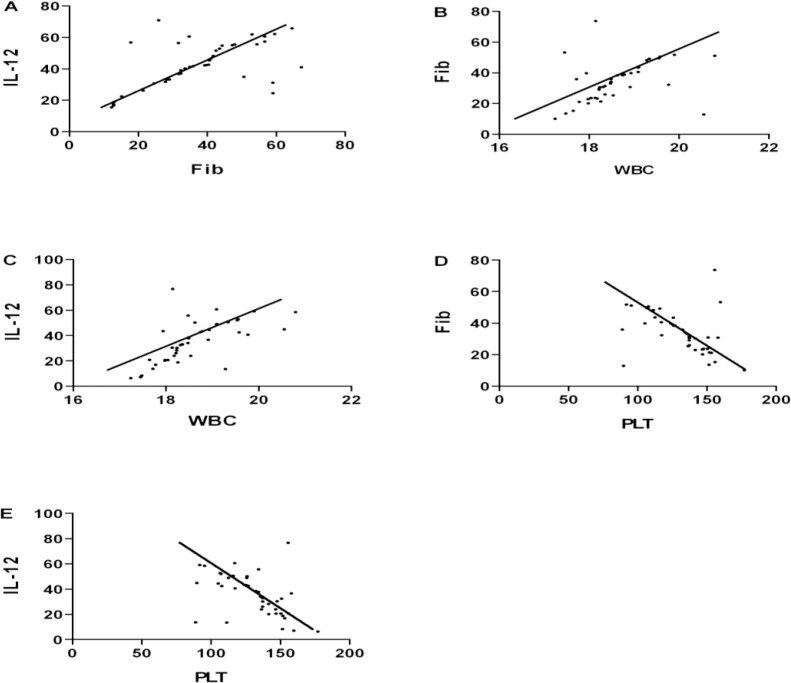
Pearson correlation analysis of serum Fib, IL-12 levels and clinical examination indexes in two groups. **(A)** There was a positive correlation between the levels of Fib and IL-12 in the serum of the children in the observation group (r=0.559, *P*<0.001). **(B)** There was a positive correlation between Fib and erythrocyte quantity (r=0.419, *P*=0.006). **(C)** There was a positive correlation between IL-12 and erythrocyte quantity (r=0.649, *P*<0.001). **(D)** There was a negative correlation between Fib and platelet quantity (r=−0.434, *P*=0.004). (E) There was a negative correlation between IL-12 and platelet quantity (r=−0.504, *P*<0.001)

There was a positive correlation between the levels of Fib and IL-12 in the serum of the children in the observation group (r=0.559, *P*<0.001) (Fig. 2A).

The expression levels of Fib, IL-12 were not significantly related to the sex and age of the children in the observation group, but related to the symptoms of vomiting, diarrhea, blood stool and slow heart rate, all of which were statistically significant (*P*<0.05) (Table 3).

**Table 3:** Relationship between clinical features and the expressions of Fib, IL-12 in observation group

***Clinicopathological parameters***	***n***	***Fib***	***t***	***p***	***IL-12***	***t***	***P***
Age (days)			0.016	0.988		0.088	0.930
≤17.1	20	35.77±14.39			40.72±14.63		
>17.1	22	35.84±14.25			40.32±14.66		
Sex			0.006	0.996		0.041	0.968
Male	29	35.96±16.31			40.62±14.69		
Female	13	35.93±15.37			40.42±14.71		
Vomiting			2.075	0.045		2.164	0.037
Yes	13	38.34±15.32			43.93±14.53		
No	29	29.53±11.43			35.53±10.14		
Diarrhea			2.059	0.046		2.082	0.044
Yes	17	37.43±15.63			43.34±14.24		
No	25	28.43±12.63			35.39±10.52		
blood stool			2.041	0.048		2.112	0.041
Yes	14	38.23±15.73			43.27±14.87		
No	28	29.43±11.74			34.43±11.65		
Slow heart rate			2.090	0.043		2.026	<0.050
Yes	9	37.73±15.79			43.63±15.34		
No	33	28.42±10.63			34.24±11.45		

The erythrocyte level in the observation group was significantly higher than that in the control group, and the platelet level was significantly lower than that in the control group (*P*<0.05). There was no significant difference in neutrophils and lymphocytes between the two groups (Table 4).

**Table 4:** Comparison of common clinical examination indexes between the two groups

***Group***	Erythrocyte(^*^10^9^/L )	Platelet(^*^10^9^/L )	***Neutrophil (%)***	***Lymphocyte (%)***
Observation group (n=42)	18.52±0.79	134.56±20.52	0.63±0.16	0.30±0.12
Control group (n=40)	4.43±0.53	239.42±21.23	0.62±0.13	0.29±0.11
*t*	94.360	22.740	0.310	0.393
*P*	<0.001	<0.001	0.758	0.696

The results of Pearson correlation analysis showed that erythrocytes were positively correlated with Fib (r=0.419, *P*=0.006), erythrocytes were positively correlated with IL-12 (r=0.649, *P*<0.001), platelets were negatively correlated with Fib (r=−0.434, *P*=0.004), and platelets were negatively correlated with IL-12 (r=−0.504, *P*<0.001). (Fig. 2B–2E).

## Discussion

NEC is clinically commonly seen in premature, low body mass infants. Children with NEC may have a history of ischemia and hypoxia or improper feeding. Abdominal distension and feeding intolerance in early clinical manifestations were mostly nonspecific. The main clinical symptoms in the middle and late stage were abdominal distension, even developed to intestinal infarction, blood stool and so on (15). Many reasons lead to ischemic and anoxic damage of intestinal mucosa, resulting in diffuse or localized functional necrosis of barrier structure in small intestine and colon, which together with bacterial invasion were considered the main link in the pathogenesis of NEC (16, 17). At present, the problems faced by NEC in prevention and treatment are thorny, so it is urgent to find the indicators that can be used to predict the diagnosis and treatment of NEC. The purpose of this study was to explore the expressions of Fib, IL-12 in children with NEC to find out more directions for the diagnosis and treatment of NEC.

The results of this study showed that the serum Fib, IL-12 in the observation group were significantly higher than those in the control group. There was a positive correlation between serum Fib and IL-12 levels in the observation group. It suggested that the expressions of Fib, IL-12 in serum of children with NEC can reflect the changes of NEC, and usually increased with the increasing of the severity of the disease. Fibrin peptide A (FPA) released by Fib increased to 60% in patients with inflammatory bowel disease (18). The expressions of IL-12 and mRNA secreted by monocytes in patients with ulcerative colitis was significantly higher than that in patients with non-active or normal colitis (19), consistent with the results of our study.

The results showed that the expression levels of Fib, IL-12 were not significantly related to the sex and age of the children in the observation group, but related to the symptoms of vomiting, diarrhea, blood stool and slow heart rate. It suggested that with the different clinicopathological features of NEC, Fib, IL-12 had different expression levels, and the manifestations, such as diarrhea and blood stool, may be related to inflammatory changes in NEC patients. B cells, which promote the secretion of IL-12, could cause intestinal inflammation (20), and IL-12p40 in colorectal cells could induce allergic diarrhea (21). There was also a correlation between Fib and the value of activity index of inflammatory bowel disease (22). All the above studies show that Fib and IL-12 can cause intestinal inflammatory changes in NEC, and affect the stress response of patients such as vomiting and diarrhea by regulating changes in the body’s inflammatory response.

Generally speaking, there is little literature on the relationship between the abnormal expressions of Fib, IL-12 and the clinical features of NEC. To explore how the abnormal expressions of Fib, IL-12 can cause abnormal inflammatory changes in the body and cause the symptoms such as vomiting, diarrhea, blood stool, slow heart rate and so on, is one of the key research directions in the later stage. Serum biological related biochemical markers have been widely used in clinic because of their small trauma and no radiation injury. In this study, the erythrocyte level in the observation group was significantly higher than that in the control group, and the platelet level was significantly lower than that in the control group. There was no significant difference in neutrophils and lymphocytes between the two groups.

Therefore, children with abnormal erythrocyte and platelet levels are at higher risk of developing severe NEC. From the correlation analysis, the expressions of Fib and IL-12 were positively correlated with WBC, negatively correlated with PLT, and not correlated with neutrophils and lymphocytes. The results showed that the levels of WBC, PLT were correlated with the expressions of Fib and IL-12, which further confirmed that Fib and IL-12 could be used as reference indexes to judge the prognosis of children. Recombinant human erythropoietin (rhEPO) promoted the proliferation and development of intestinal microvascular endothelial cells in children, and formed intestinal barrier to resist pathogenic bacteria (23, 24). Children with thrombopenia, were nearly 90% likely to find intestinal necrosis in them after operation (25). It can be seen from the study that in the blood routine examination of children with NEC, the blood cell level is usually increased and the platelet is decreased.

Fib and IL-12 can reflect the inflammatory changes of NEC, which together with the blood routine have the function of inflammatory detection. It was confirmed from the side that the condition of NEC children would change with significant abnormal changes in WBC, PLT levels, and WBC, PLT levels were correlated with the expressions of Fib and IL-12.

## Conclusion

The expression level and correlation of Fib, IL-12 in serum of children with NEC and its relationship with clinicopathological features were studied. The results showed that the expressions of Fib, IL-12 in serum of children with NEC could reflect objectively and accurately the changes of NEC. However, there are still some shortcomings. For example, the expression levels of Fib, IL-12 in prognosis were not studied further. In the follow-up experiment, we will continue to explore in order to provide more sensitive index data basis for clinical treatment of NEC children.

## Ethical considerations

Ethical issues (Including plagiarism, informed consent, misconduct, data fabrication and/or falsification, double publication and/or submission, redundancy, etc.) have been completely observed by the authors.
